# Comment on: The lost art of argumentation

**Published:** 2017-06-30

**Authors:** Michael Epstein

**Affiliations:** 1 Center for Integrative Medicine, University of Saskatchewan, Saskatchewan, Canada

Leontowicz identifies the topic of argumentation as a critical competency for medical students and practicing physicians, and suggests that the topic be included as part of medical education programs.^1^ I would first like to applaud the author for identifying this topic, and I would like to contribute to the conversation with a few observations concerning argumentation as it relates to medical practice and medical leadership, with related suggestions for integration into medical education programs.

The word argumentation, as typically defined, involves the integration of two components: the first is critical/creative thinking, and the second is effective communication and messaging. Since these two components are both typically included within existing medical curricula, the topic of argumentation could involve enriched learning activities which build upon and integrate these two components.

I have one suggestion regarding definitions. The word argumentation is often equated with debate. In fact however, debate represents one important form of argumentation, but is by no means the only one. Argumentation can also be employed in less adversarial forms of discourse such as dialogue, discussion, dialectics, devil’s advocate, and others. Each of these forms of discourse have their own specific tone, purpose, and orientation, and each may be appropriate, or not, in a given social context. For example, the adversarial tone which characterizes a true debate might be appropriate in a detached academic scholarly setting, but may be completely inappropriate conversation between a doctor and a patient.

Argumentation can also be employed in constructing proposals, and in other forms of persuasive communication such as policy advocacy. So I would suggest focusing on a broad conception of argumentation, and identify debate as simply one context within which argumentation competencies can be learned and applied. Related competencies might include the ability to define each of the above forms of discourse (dialogue, discussion, and so forth) to explain how they differ, and to identify situations under which each might be appropriate.

The topic of argumentation is also related to other curriculum topics such as shared decision-making, evidence-based practice, and clinical reasoning. It may thus be possible to integrate argumentation into existing learning activities involving these closely related topics as well.

Second, the topic of argumentation as broadly conceived has a potential place in each of the seven CanMEDS roles. It may thus be useful for the purpose of comprehensive curriculum planning, to look at each of the CanMEDS roles, and to identify where and how the topic of argumentation might fit.

Third, as the author indicates, communication and messaging represent potentially powerful psychosocial interventions. A well formulated, logically correct, and accurate statement – the essence of critical thinking – no matter how well intended and how accurately delivered, always carries with it the potential to do more harm than good. The potential for argumentation to have unintended negative consequences thus needs to be recognized, and the importance of *constructive* criticism and *socially appropriate* argumentation must be respected. Implications for curriculum design could include the distinction between being *right* and being *helpful*. Curriculum design should also embody the principle that the greater the degree of cognitive/sociopolitical conflict in a given social context, the greater the need for sensitivity, compassion, and psychological safety.

Fourth, personality variables play a significant role in the way argumentation is delivered and received. Implications for medical schools could include discussions of variables such as tolerance for conflict, tolerance for ambiguity, sensitivity to criticism, and how attention to these personality traits can help ensure positive learning outcomes.

Finally, in an era where the need for deep, meaningful, transformational change in healthcare and medical systems is becoming more and more urgent, the role of argumentation in change leadership should be underscored. This could involve more deeply integrating the topic of argumentation into the collaborator, leader-manager, and advocate roles. This might be effectively accomplished through various forms of experiential learning such as participatory educational theatre, which have been shown to be effective for cultivating competencies pertaining to capacity in conversational change leadership.

Please note that these suggestions are meant to be neither definitive nor exhaustive, but rather simply to illustrate a few ways in which the topic of argumentation could be developed and effectively integrated across the full spectrum of medical and allied health professions education.

I therefore hope that the author’s letter on the lost art of argumentation will serve to start a rich and productive conversation, and thus help to give this topic the attention that it deserves, thereby supporting the development of effective clinicians, and physician-leaders, for addressing the many complex health-related challenges of the next decade.

## Further Reading

Detailed references addressing the themes discussed in this commentary are available from the author.

For an excellent review addressing the topics of strategic messaging, interpersonal influence and conversational change leadership, the following work is recommended:

Hoggan J, Litwan G. *I’m right and you’re an idiot: the toxic state of public discourse and how to clean it up.* Gabriola Island, BC, Canada: New Society Publishers, 2016.
